# A novel *in vitro* metric predicts *in vivo* efficacy of inhaled silver-based antimicrobials in a murine *Pseudomonas aeruginosa* pneumonia model

**DOI:** 10.1038/s41598-018-24200-w

**Published:** 2018-04-23

**Authors:** Parth N. Shah, Kush N. Shah, Justin A. Smolen, Jasur A. Tagaev, Jose Torrealba, Lan Zhou, Shiyi Zhang, Fuwu Zhang, Patrick O. Wagers, Matthew J. Panzner, Wiley J. Youngs, Karen L. Wooley, Carolyn L. Cannon

**Affiliations:** 1 Department of Microbial Pathogenesis and Immunology, Texas A & M Health Science Center, College Station, TX 77843 United States; 2 Department of Chemistry, Department of Chemical Engineering, Department of Materials Science and Engineering, and Laboratory for Synthetic-Biologic Interactions, Texas A & M University, College Station, TX 77842 United States; 3 Tashkent Paediatric Medical Institute, Tashkent, 100140 Uzbekistan; 4 Department of Pathology, University of Texas Southwestern Medical Center, Dallas, TX 75390 United States; 5 Department of Chemistry and Center for Silver Therapeutics Research, The University of Akron, Akron, OH 44325 United States; 6 Department of Statistics, Texas A & M University, College Station, TX 77842 United States

## Abstract

To address the escalating problem of antimicrobial resistance and the dwindling antimicrobial pipeline, we have developed a library of novel aerosolizable silver-based antimicrobials, particularly for the treatment of pulmonary infections. To rapidly screen this library and identify promising candidates, we have devised a novel *in vitro* metric, named the “drug efficacy metric” (DEM), which integrates both the antibacterial activity and the on-target, host cell cytotoxicity. DEMs calculated using an on-target human bronchial epithelial cell-line correlates well (R^2^ > 0.99) with *in vivo* efficacy, as measured by median survival hours in a *Pseudomonas aeruginosa* pneumonia mouse model following aerosolized antimicrobial treatment. In contrast, DEMs derived using off-target primary human dermal fibroblasts correlate poorly (R^2^ = 0.0595), which confirms our hypothesis. SCC1 and SCC22 have been identified as promising drug candidates through these studies, and SCC22 demonstrates a dose-dependent survival advantage compared to sham treatment. Finally, silver-bearing biodegradable nanoparticles were predicted to exhibit excellent *in vivo* efficacy based on its *in vitro* DEM value, which was confirmed in our mouse pneumonia model. Thus, the DEM successfully predicted the efficacy of various silver-based antimicrobials, and may serve as an excellent tool for the rapid screening of potential antimicrobial candidates without the need for extensive animal experimentation.

## Introduction

*Pseudomonas aeruginosa*, a common gram-negative opportunistic pathogen, causes an estimated 51,000 serious healthcare-associated infections annually in the United States, and has been assigned a threat level of “serious” by the CDC^1^. Approximately 15% of these infections are caused by multidrug-resistant *P. aeruginosa* (MDR-PA), which are associated with worse overall clinical outcomes^1,2^. In particular, *P. aeruginosa* is one of the greatest causes of morbidity and mortality in pulmonary infections, such as ventilator-associated pneumonia (VAP) and chronic lung infections in patients suffering from cystic fibrosis (CF)^3,4^. The eradication of *P. aeruginosa* infection is particularly challenging due to its high degree of innate resistance to many antibiotic classes coupled with its ability to readily acquire additional resistance^3,5,6^. Similarly, other bacterial pathogens are also acquiring resistance rapidly, thereby turning antibiotic resistance in to a serious and growing global problem. Thus, to combat this problem, development of novel classes of antimicrobials is urgently required.

Silver (Ag) is particularly attractive as an antimicrobial candidate due to its excellent potency, broad spectrum activity, and low toxicity towards eukaryotic cells^7–10^. Few accounts of silver resistance have been reported and in most cases including *P. aeruginosa*, resistance to silver is transient and unstable, despite its widespread and continuous use^11–13^. Even though true, stable, silver resistance imparted through acquisition of plasmids or transposons has been documented in *Salmonella* Typhimurium and *Escherichia coli*^14–16^, few such strains exist, and no known reports of transmission of these components to *P. aeruginosa* exist^17^. However, poor bioavailability remains a significant challenge, because Ag^+^ cation, the bioactive form of silver, readily reacts with a wide range of physiological substrates and is rapidly rendered inactive^11^. Youngs *et al*. have synthesized N-heterocyclic silver carbene complexes (SCCs) with a diverse set of chemical structures and physico-chemical properties to overcome this challenge^18–24^. These SCCs provide gradual release of bioactive Ag^+^ from a stable silver complex, thus enabling retention of antimicrobial activity for prolonged periods in physiological conditions and sustaining its bioavailability^19,20^. For instance, the methylated caffeine-based SCC1 has served as one of the main candidates of interest, due to its moderate aqueous solubility and low toxicity^21^. SCCs derived using an imidazolium cation-based backbone, such as 4,5-dichloroimidazole, have also been synthesized due to their greater stability in aqueous solutions^22^; among these, some have been designed to be highly water soluble, such as SCC22 (up to 110 g/L)^23^, while others are highly lipophilic, such as SCC23 and SCC28^25^. Each of these SCCs has demonstrated broad spectrum antimicrobial activity against a diverse range of pathogenic bacteria *in vitro*^18–21,23–26^.

Our interest in the development of antimicrobials for the treatment of pulmonary bacterial infections has led us to explore aerosolization of SCCs, because this delivery method achieves higher local concentrations of drug in the lungs with reduced concern for systemic toxicity^27^. Our preliminary *in vivo* investigation has demonstrated the efficacy of SCC1 in a murine acute *P. aeruginosa* lung infection model^21^. Yet, an improvement in the pharmacokinetics of small molecule therapeutics such as the SCCs, delivered to the lung, can be achieved by employing formulations capable of providing sustained delivery at the target site^27,28^. Our previous work with SCC-loaded, L-tyrosine polyphosphate (LTP) and shell cross-linked knedel-like (SCK) nanoparticle formulations (NPs) for the treatment of *P. aeruginosa* lung infections has shown a survival advantage compared with sham treatment in our acute *P. aeruginosa* infection model with SCC doses far lower than those required of free drug to achieve the same clinical effect^29,30^.

While we have demonstrated efficacy of several silver-bearing antimicrobial formulations *in vivo*, we have explored only a small subset of the total possible formulations. The recent development of biodegradable polyphosphoester-based SCK NPs^31^ has further increased the number of available platforms for SCC delivery to the lung^32^. Therefore, a rational approach to identify promising candidates for *in vivo* efficacy and safety testing is needed. “Therapeutic index” (TI), a combined quantitative metric based on the relative *in vivo* safety and efficacy of a compound, appears to provide a convenient method for the identification of promising therapeutic candidates^33^. In this study, we have developed an *in vitro* adaptation of the therapeutic index, named the drug efficacy metric (DEM), which is effectively the ratio of the drug toxicity breakpoint (*in vitro* half-median lethal dose, i.e., LD_50_) and the drug potency breakpoint (*in vitro* minimum bactericidal concentration, i.e., MBC), i.e., LD_50_/MBC. Because our goal was to identify *in vitro* parameters that are predictive of *in vivo* antimicrobial efficacy, we have correlated *in vitro* metrics such as the DEM, minimum inhibitory concentration (MIC), MBC, and LD_50_ with median survival hours (a metric for *in vivo* efficacy) of *P. aeruginosa* infected mice following aerosolized treatment with SCCs. We hypothesized that the DEM would correlate the best with *in vivo* efficacy compared with other metrics such as the MIC, MBC, and LD_50_ alone and that SCCs with higher *in vitro* DEM values would demonstrate superior *in vivo* efficacy. Next, to validate our hypothesis that an on-target cell type is critical for the DEM to accurately predict *in vivo* efficacy, we have calculated the DEM using a human bronchial epithelial cells 16HBE (on-target) and primary human dermal fibroblasts HDFs (off-target) and correlated both DEMs to median survival hours. Next, *in vivo* dose-response studies were performed using the two most promising SCCs (SCC1 and SCC22) with the highest DEM to investigate whether increased doses of the SCCs lead to a better survival outcome. Lastly, we validate the predictive ability of the DEM, by demonstrating that the DEM is capable of predicting the *in vivo* efficacy of a silver-bearing nanoparticle formulation defined as median survival hours.

## Results

### *In vitro* testing of silver-based antimicrobial formulations and determination of *in vitro* Drug Efficacy Metric (DEM)

**Figure 1:**
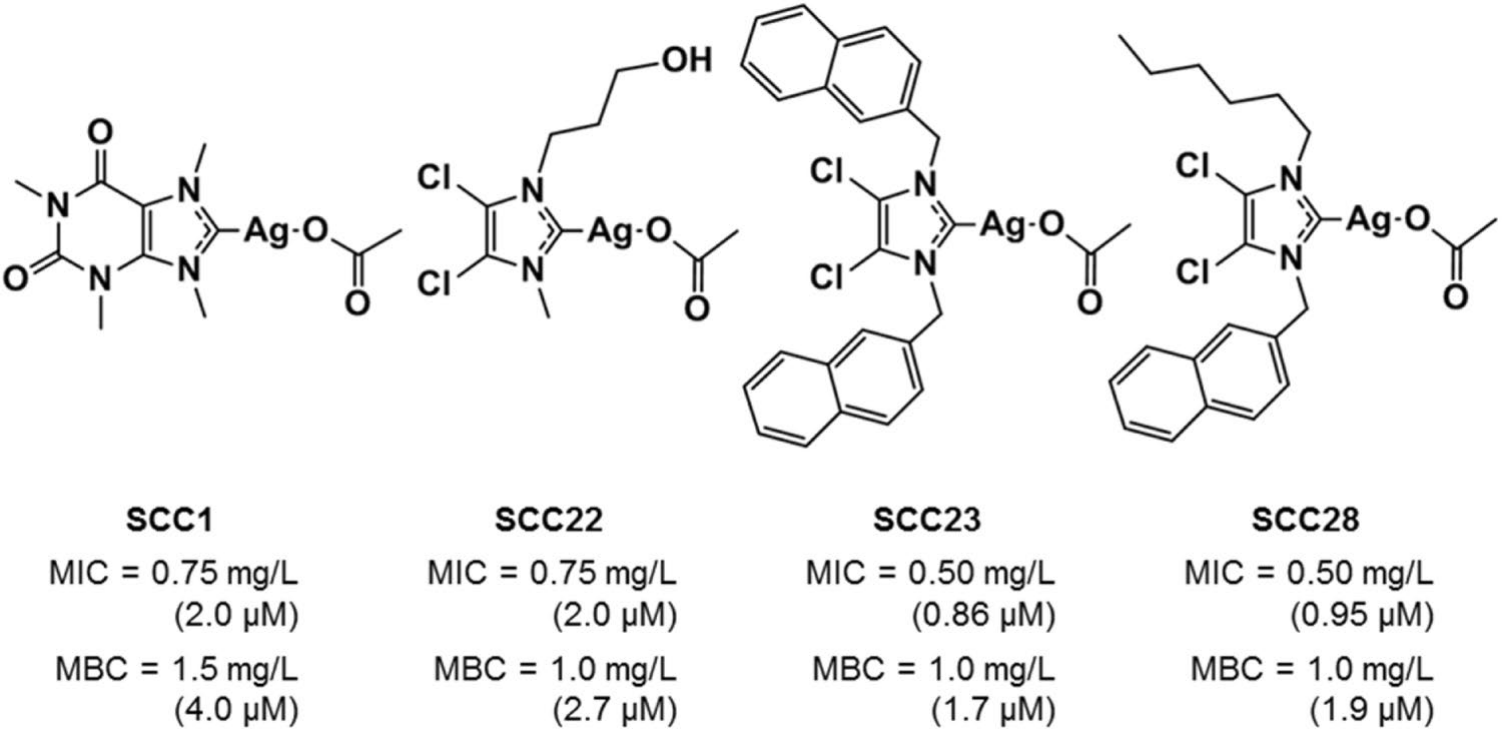
Chemical structures of select N-heterocyclic silver carbene complexes (SCCs) and their antimicrobial activity against a mucoid clinical *P. aeruginosa* strain PA M57-15. The SCCs studied are based on xanthines (SCC1) and 4,5-dichloroimidazoles (SCC22, SCC23, and SCC28).

The chemical structures of the four SCCs investigated in this manuscript and a summary of their antimicrobial activity is presented in Fig. 1. Initially, preliminary cytotoxicity screening of a subset of eight SCCs from a library of ~30 SCCs was performed using alamarBlue^®^ against immortalized human bronchial epithelial cells (16HBE) to determine *in vitro* LD_50_ (Supplementary Figure S1). The four SCCs discussed here were chosen based on their divergence of chemical structures and *in vitro* cytotoxicity profile against 16HBEs. These four SCCs were evaluated for their antimicrobial activity against a representative mucoid clinical *P. aeruginosa* strain, PA M57-15, by determining their MIC and MBC. The hydrophobic, naphthalene containing 4,5-dichloroimidazole based SCC23 and SCC28 have comparable MICs against PA M57-15 on a molar basis (0.86 and 0.95 µM, respectively), while hydrophilic SCC22 and SCC1 have a slightly higher MIC at 2 µM (Fig. 1). With respect to the MBCs, even though SCC1 had the highest MBC (4 µM) and SCC23 had the lowest MBC (1.7 µM) on a molar basis, the MBCs for all four SCCs are almost identical and within experimental error on a µg/mL basis. Evaluation of Ag-PPE-SCKs against 16HBEs demonstrated an LD_50_ of 100 µM; whereas, the MBC against PA M57-15 was 0.188 µg/mL, based on silver mass, which is equivalent to that of SCC23 on a molar basis (~1.7 µM).

**Figure 2:**
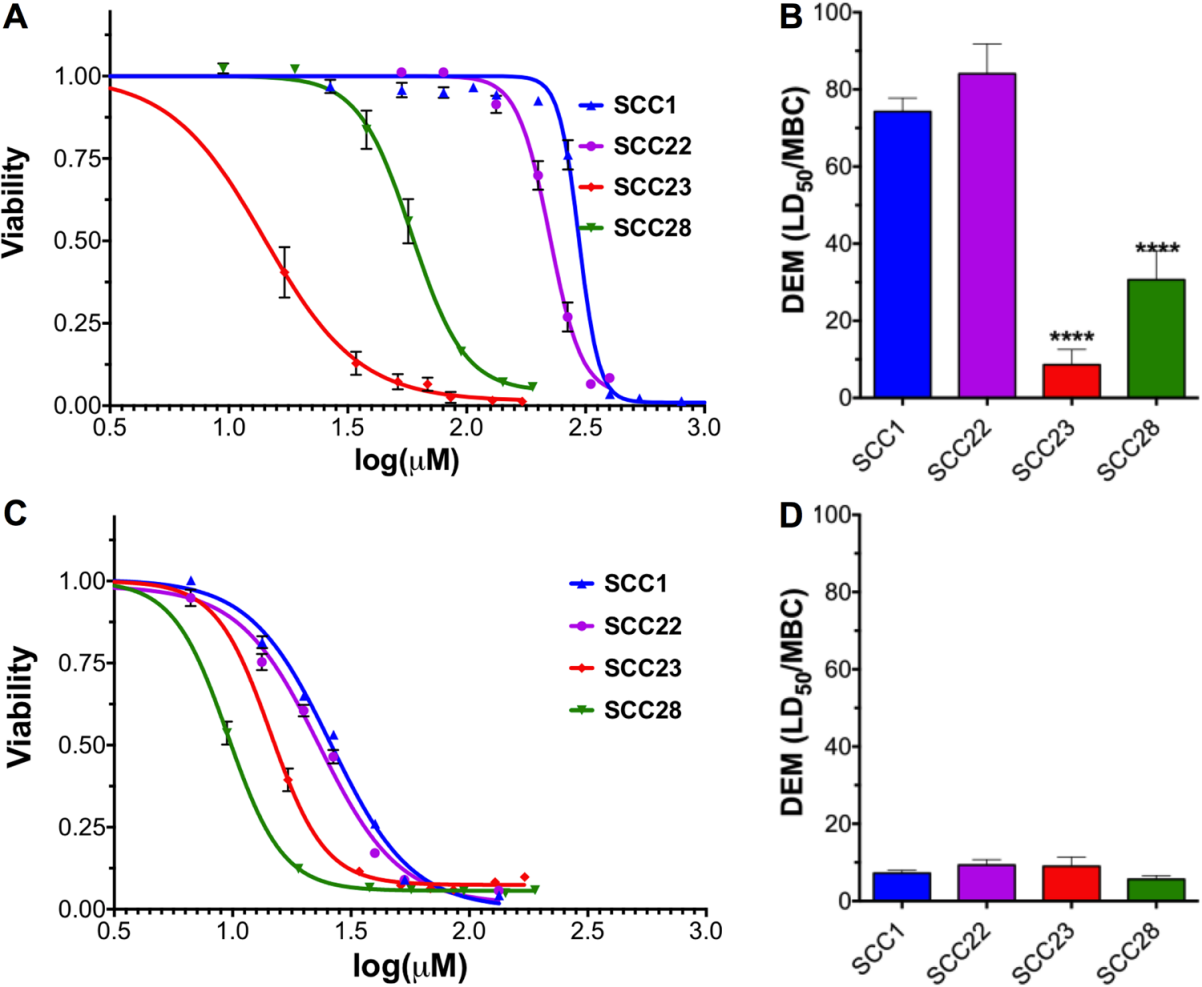
Cell viability curves of SCCs against (**A**) 16HBE cells and (**C**) HDFs and the corresponding DEMs (LD_50_/MBC) of SCCs against (**B**) 16HBE cells and (**D**) HDFs. A total of 12 replicates were performed (3 technical replicates × 4 biological replicates = 12 total replicates) and the data are presented as mean ± standard error of the mean. Statistical analysis was performed using ordinary one-way ANOVA with Tukey’s multiple comparisons test, where ****P ≤ 0.0001.

Based on the alamarBlue^®^ cytotoxicity assay using 16HBEs, many of the SCCs have similar toxicity profiles; however, SCC23 and SCC28 based on a 4,5-dichloroimidazole backbone have notably higher toxicity with LD_50_ values of 14.7 and 58.1 µM respectively (Supplementary Figure S1 and Fig. 2A). On the other hand, SCC1, a methylated-caffeine based hydrophilic SCC has an LD_50_ of 297 µM and SCC22, a hydrophilic complex based on a 4,5-dichloroimidazole backbone has an LD_50_ of 224 µM, thus, suggesting the low toxicity of these compounds (Fig. 2A). When tested for toxicity against human dermal fibroblasts (HDFs), minimal differences were observed among the four SCCs with the LD_50_ values being 25.8, 22.7, 14.2, and 9.8 µM for SCC1, SCC22, SCC23, and SCC28, respectively (Fig. 2C). Furthermore, the LD_50_ values for SCC23 are comparable for 16HBEs and HDFs, while significant differences exist between the LD_50_ values for the two cell types with regards to the other SCCs. Finally, the Ag-PPE-SCKs were only tested against 16HBEs, yielding an average LD_50_ of 100 µM.

The DEM, which is the ratio of the *in vitro* LD_50_ to the *in vitro* MBC, was subsequently calculated for each SCC and Ag-PPE-SCKs. When comparing the SCCs *via* the DEM using 16HBEs, an on-target cell type, significant differentiation between the compounds was seen; SCC1 and SCC22 have significantly higher DEMs than SCC23 and SCC28 (Fig. 2B) as determined by ordinary one-way ANOVA with Tukey’s multiple comparisons test. However, SCC22 is not significantly higher in DEM than SCC1. Thus, the ranking of the SCCs determined by16HBE-based DEM values is SCC22 > SCC1≫ SCC28 > SCC23. Furthermore, the DEM of Ag-PPE-SCKs determined using 16HBEs is slightly lower than SCC1, but the difference is non-significant (P = 0.0528) as determined by unpaired t-test (Fig. 6). In contrast, when using HDFs, an off-target cell type, no distinct trends could be distinguished. SCC23 and SCC22 show slight and non-significant trends for the highest DEM (Fig. 2D), yet no significant differences can be found between DEM values for any of the SCCs (P > 0.05).

### Aerosolized SCC22 and SCC1, but not SCC23 and SCC28, are efficacious *in vivo* in a mouse model of acute *P. aeruginosa* pneumonia

**Figure 3:**
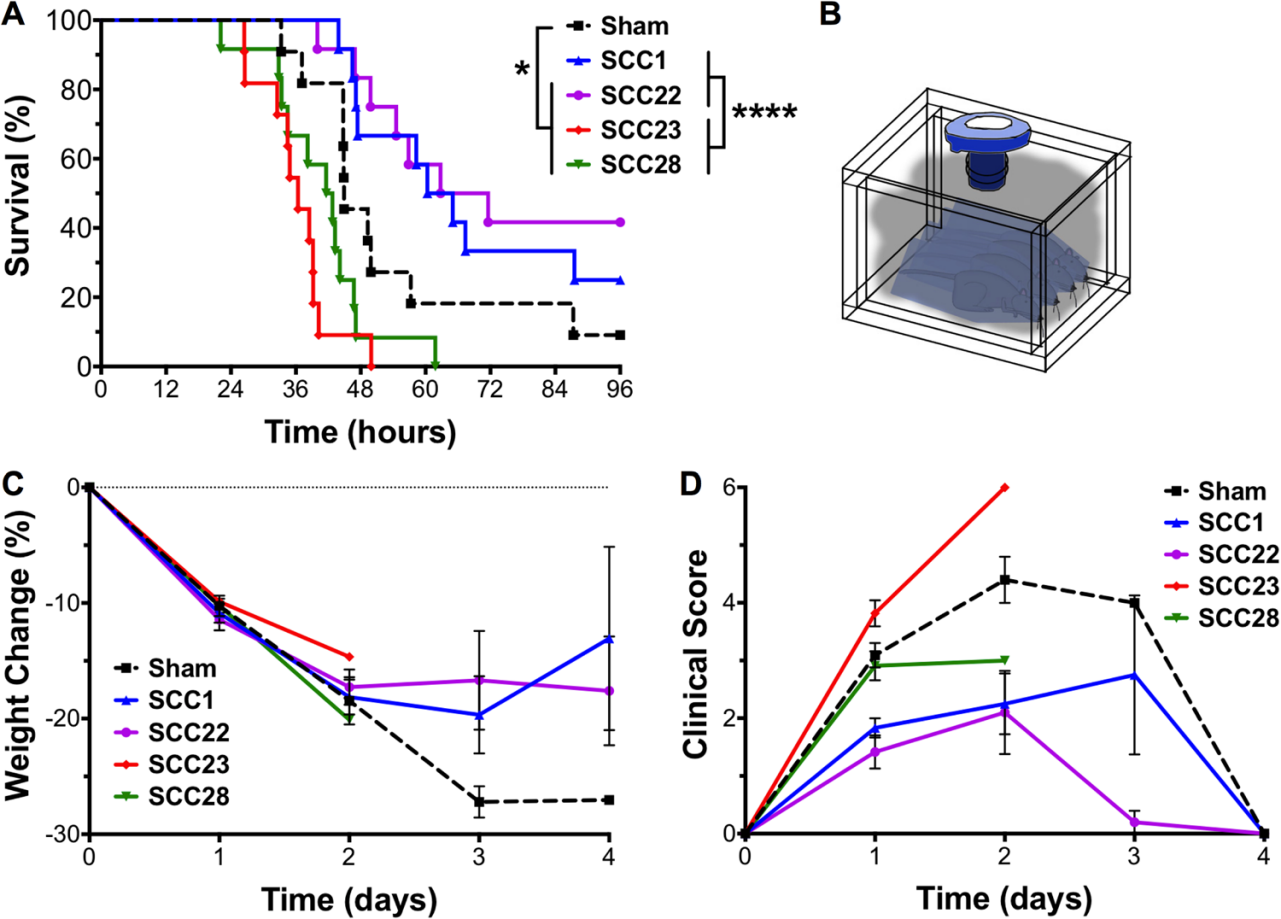
(**A**) Survival curves demonstrating the efficacy of aerosolized SCCs compared with sham treatment in an acute *P. aeruginosa* lung infection model, where *P ≤ 0.05; ****P ≤ 0.0001, as determined by log-rank Mantel-Cox test. The mice were administered aerosolized treatments in a nose-only fashion by placing in CH-247 restraint tubes and then in the multi-dosing chamber as illustrated in the schematic (**B**). (**C**) Weight loss and (**D**) average daily clinical score in mice following infection and SCC treatment. Six mice were used per treatment arm for each experiment and the data shown are pooled from two independent experiments.

The antimicrobial efficacy of SCC1, SCC22, SCC23, and SCC28 have been tested side-by-side as nebulized treatments in a mouse model of acute *P. aeruginosa* lung infection. Of the 25 μmol of the SCCs delivered into the multi-dosing chamber, approximately 1% is delivered to the lungs of each mouse as demonstrated by our previous biodistribution data^34,35^, which results in an effective silver (Ag^+^) dose of ~1.3 mg/kg or 27.3 μg per mouse per dose. The mice treated with SCC22 resulted in a superior survival of 42% compared to sham (9.1%, P ≤ 0.05), while those treated with SCC1 demonstrated a 25% survival, thus demonstrating a survival trend compared with sham treatment (P = 0.0831) (Fig. 3A). In contrast, mice treated with SCC23 and SCC28 had significantly lower survival compared with mice administered sham treatment (P ≤ 0.05), as well as mice administered SCC22 and SCC1 treatments (P ≤ 0.001; Fig. 3A). None of the treatments had a significant effect on mouse weight throughout the 96 hours of the study (Fig. 3C), though the SCC1 and SCC22 treated mice showed significantly lower signs of illness at 24 hours following infection (P = 0.0378 and 0.0060, respectively) (Fig. 3D). Furthermore, the mice treated with SCC23 appeared the most infirm at each time-point based on the clinical illness scores. However, the investigators assigning the scores were not blinded to the treatment group, which is a limitation of this study.

### Histological examination of lung tissue following aerosolized treatment with SCCs does not adequately correlate with *in vivo* outcome

**Figure 4:**
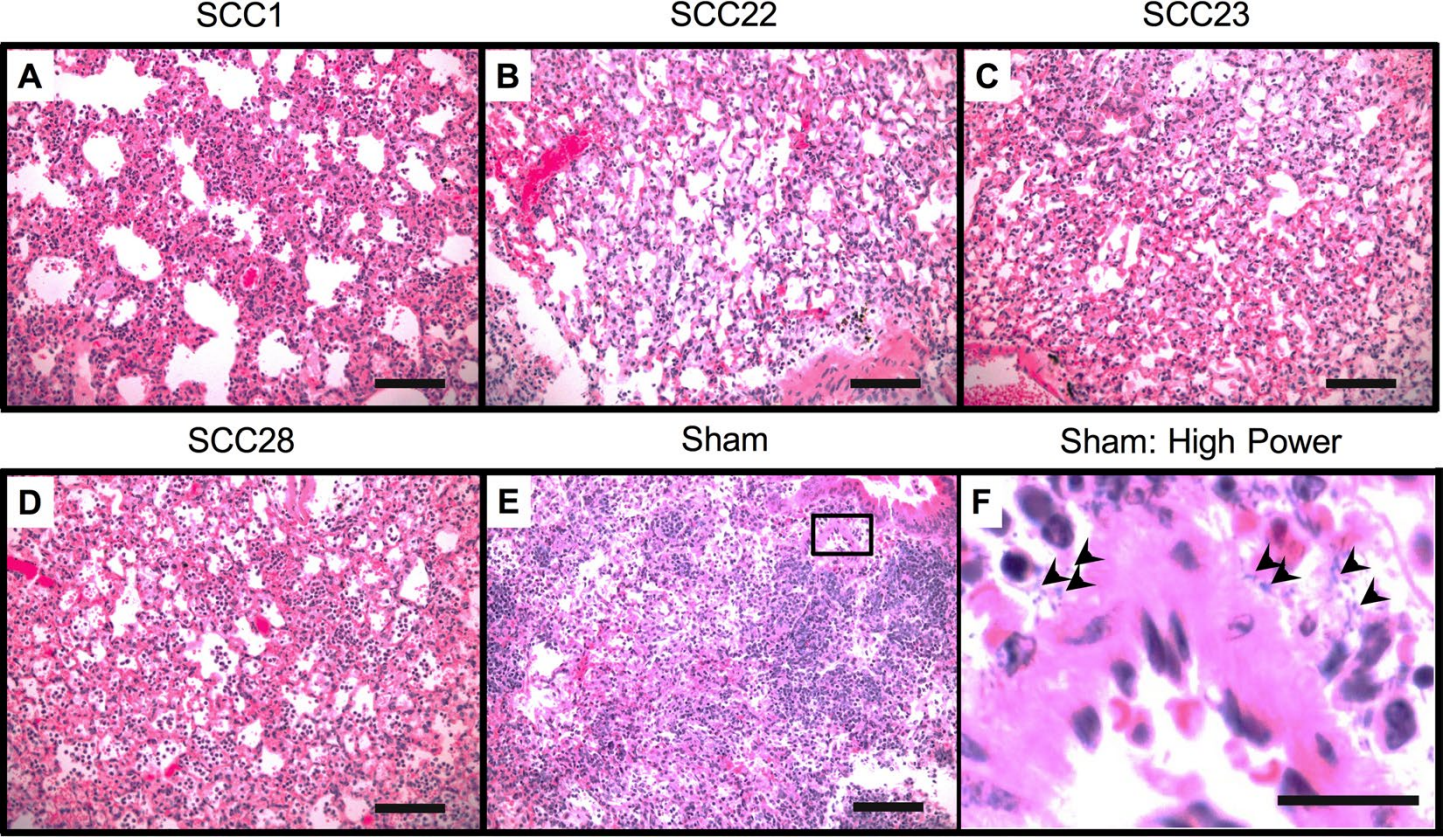
Representative photomicrographs demonstrating differences in lung morphology of mice infected with *P. aeruginosa* strain PA M57-15 and treated with (**A**) SCC1, (**B**) SCC22, (**C**) SCC23, (**D**) SCC28, and (**E**,**F**) sham treatment. The abundant presence of bacterial colonies in the lungs of sham-treated animals is highlighted with arrows in (**F**). Bacterial colonies were not observed in the lungs of any animals treated with SCCs (data not shown). Images (**A**) through (**E**) taken at 20X and (**F**) taken at 60X and corresponds to the section in (**E**) marked with a box. Scale bars for (**A**) through (**E**) = 100 μm and scale bar for (**F**) = 20 μm.

Histological examination of the lungs of infected animals treated with nebulized SCCs or vehicle (sham) allowed differentiation into three tiers of increasingly abnormal appearance, but was unable to provide additional insights into the *in vivo* survival outcome. The lungs of mice treated with SCC22 (Fig. 4B) appeared less inflamed and abnormal than those of SCC1 (Fig. 4A), SCC23 (Fig. 4C) and SCC28 (Fig. 4D), which were less abnormal than the lungs of sham-treated mice (Fig. 4E,F). As expected, the lungs of vehicle-treated animals show dense, diffuse, and severe inflammatory infiltrate in both interstitial and intra-alveolar spaces with extensive areas of acute consolidation consistent with pneumonia. Furthermore, severe congestion and haemorrhage was also observed and consolidated areas also reveal abundant bacterial colonies. These mice exhibited an aggregate histological score of +6 based on inflammation, consolidation, and haemorrhaging as parameters. In contrast, lungs of animals treated with SCC22 revealed mild, patchy interstitial mononuclear and polymorphonuclear inflammation with small, focal areas of parenchymal neutrophilic consolidation consistent with pneumonia. Even though vessels show mild congestion, no focal haemorrhage was observed and mice in this group had an aggregate histological score of +3. No bacterial colonies were identified in the tissue samples. Similarly, in the case of animals treated with SCC1, lung parenchyma shows mild-to-moderate inflammatory infiltrates with both mono- and polymorphonuclear cells and mild, patchy consolidation with complete absence of bacterial colonies. However, focal haemorrhages were identified along with mild vessel congestion and the mice in this group had an aggregate histological score of +4. In the case of SCC23 treated animals, mild-to-moderate polymorphonuclear neutrophilic inflammation in both interstitial areas and intra-alveolar spaces along with patchy, moderate consolidation, as well as mild congestion and haemorrhages were observed. These observations resulted in an aggregate histological score of +4 in the SCC23 treated mice. Of all SCC treatments, lungs of animals treated with SCC28 showed the highest degree of inflammation; a denser, moderate inflammatory interstitial and intra-alveolar infiltrate with predominantly polymorphonuclear neutrophils was observed. Additionally, patchy, moderate areas of consolidation and moderate parenchymal haemorrhages were also present. However, based on our semi-quantitative histopathological scoring system, the mice in this group had an aggregate score of 4, which is not different from the SCC1 and SCC23 groups. Nevertheless, bacterial colonies were not identified in the lung tissues of animals treated with either SCC23 or SCC28. Thus, histological scores allowed ranking of SCC efficacy as SCC22 > SCC1, SCC23, SCC28≫ sham.

### Drug Efficacy Metric (DEM) derived using an on-target cell type provides the best measure of *in vivo* activity for silver-based antimicrobials

A Cox proportional hazards regression model was used to identify the *in vitro* parameter that demonstrates the best correlation with the probability of survival. All *in vitro* parameters, except HDF DEM (P = 0.0835), have a significant effect on survival (P < 0.0001). The R2 values listed in Table 1 demonstrate 16HBE DEM (R2 = 0.5166) to be the most effective predictor of survival probability of all the *in vitro* parameters tested in this study.

**Table 1:** Log hazard rates and R squared values for correlation between *in vitro* parameters of antimicrobial efficacy and toxicity and probability of survival determined using a Cox proportional hazards regression model.

*In vitro* Parameter	Log Hazard Rate (p-value)	R squared
MIC	−2.0282 (<0.0001)	0.5014
MBC	−1.1206 (0.0001)	0.3269
16HBE LD_50_	−0.0093 (<0.0001)	0.4646
16HBE DEM (LD_50_/MBC)	−0.0335 (<0.0001)	0.5166
HDF LD_50_	−0.1220 (<0.0001)	0.4065
HDF DEM (LD_50_/MBC)	−0.2089 (0.0835)	0.0617

**Figure 5:**
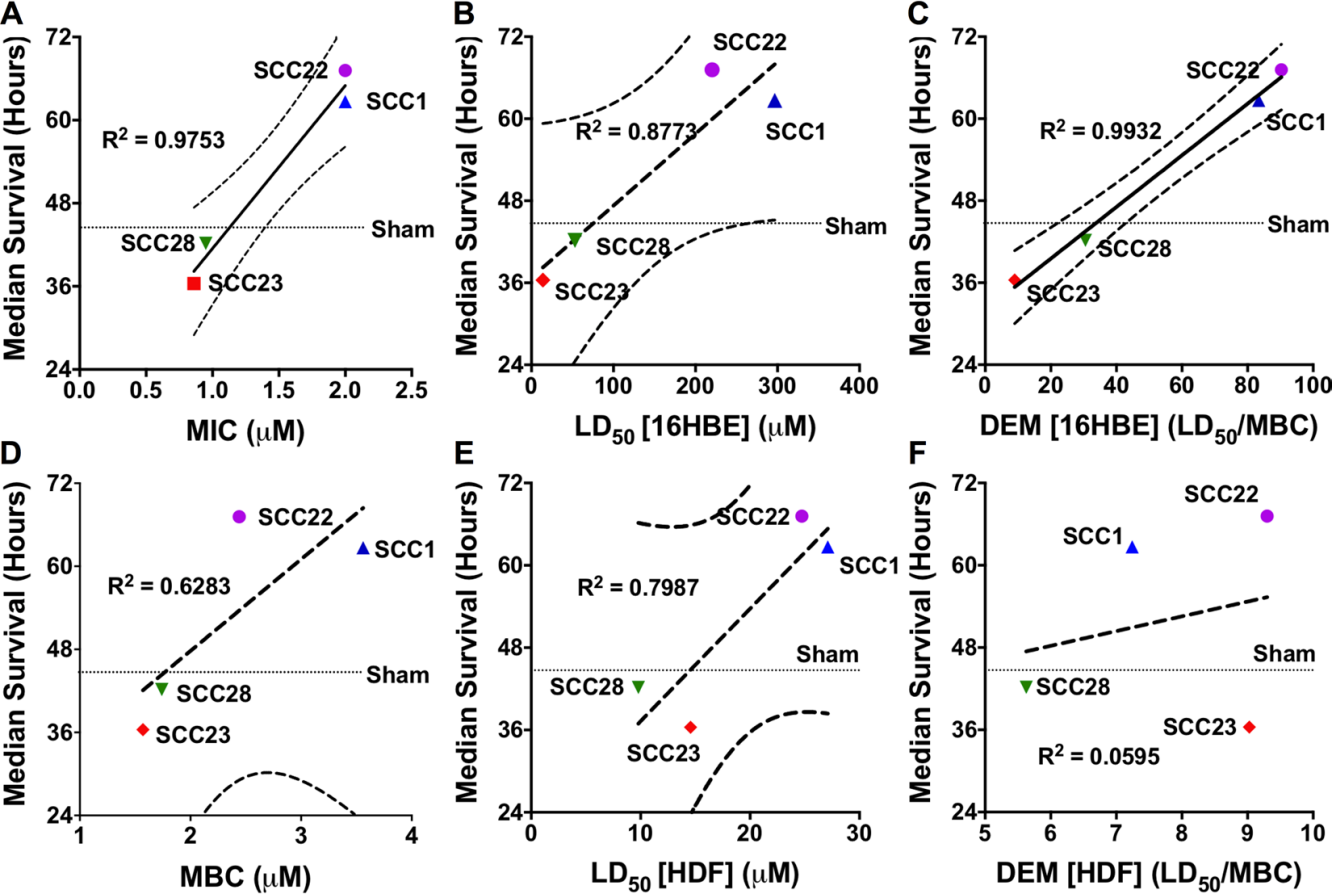
Correlation between mouse median survival after infection with PA M57-15 and treatment with aerosolized SCCs and (**A**) MIC against PA M57-15, (**B**) LD_50_ against 16HBEs, (**C**) the DEM calculated using cytotoxicity vs. 16HBEs, (**D**) MBC against PA M57-15, (**E**) LD_50_ against HDFs, and (**F**) DEM calculated using cytotoxicity vs. HDFs.

We have also utilized linear regression to correlate the median survival hours for the mice by SCC treatment with *in vitro* parameters for antimicrobial activity and toxicity, namely, MIC and MBC against PA M57-15, LD_50_ against eukaryotic cells (16HBEs and HDFs), and the DEM (LD_50_/MBC). Cell cytotoxicity, as measured by LD_50_ against the 16HBE cells, was fairly predictive of *in vivo* efficacy (R^2^ = 0.8773) (Fig. 5B) and SCCs with lower toxicity generally resulted in higher efficacy. On the other hand, cytotoxicity against HDFs was not as well correlated with survival (R^2^ = 0.7987) (Fig. 5E). When using antimicrobial potency, good correlation between *in vitro* MIC and *in vivo* efficacy was observed (R^2^ = 0.9753); however, the correlation with *in vitro* MBC was poor (R^2^ = 0.6283) (Fig. 5A,D). Furthermore, these correlations appear to be in a reverse order with less potent SCCs generally resulting in better survival. For instance, SCC23 has the lowest MBC (1.7 μM) of all 4 SCCs against PA M57-15, yet it yielded the worst survival outcome. On the other hand, SCC1 has the highest MBC (4.0 μM) among the 4 SCCs tested, yet it resulted in a survival advantage over sham treatment. When using the DEM, a combined metric based on *in vitro* antimicrobial activity (MBC) against PA M57-15 and *in vitro* cytotoxicity against 16HBEs, the best correlation with *in vivo* efficacy was observed (R^2^ = 0.9932, Fig. 5C). The correlative equation resulting from linear regression of this data is Y = 0.3777X + 31.97, where Y = median survival hours and X = mean DEM. Furthermore, as expected, the SCCs with higher DEMs resulted in superior *in vivo* efficacy. Interestingly, when 16HBEs were substituted with HDFs for cytotoxicity determination, however, the DEM correlation was very poor (R^2^ = 0.0595, Fig. 5F) underscoring the importance of the on-target cell type for DEM determination.

### DEM predicts the *in vivo* efficacy of aerosolized Ag-PPE-SCKs

**Figure 6:**
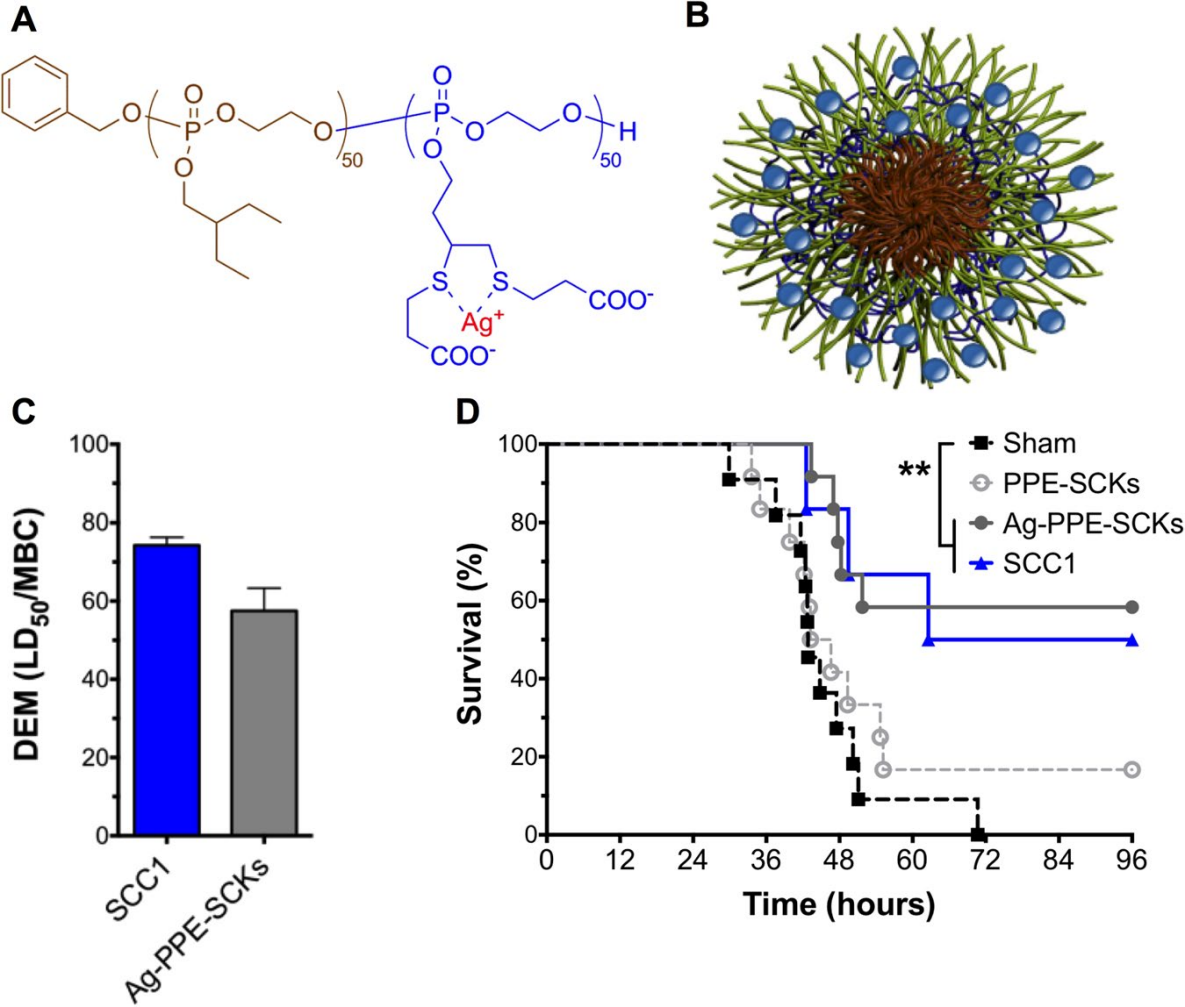
(**A**) Chemical structure of the polyphosphoester polymer (PPE) utilized to formulate Ag-PPE-SCK nanoparticles, (**B**) Schematic representation of Ag-PPE-SCK NPs, (**C**) Comparison of the DEMs of SCC1 and the Ag-PPE-SCKs, as calculated using LD_50_ against 16HBEs, and (**D**) mouse survival post-infection after aerosolized treatment with SCC1, unloaded PPE-SCKs, or Ag-PPE-SCKs. **P ≤ 0.01 as determined by log-rank Mantel-Cox test.

SCC1 and Ag-PPE-SCKs were compared head-to-head in our acute *P. aeruginosa* lung infection model due to their similar and statistically indifferent *in vitro* DEMs. Based on the DEM value for Ag-PPE-SCKs (mean DEM = 58.82), we predicted the median survival of mice in that group to be 54.2 hours, which translates to a survival of 50% of the cohort at 54.2 hours theoretically. In our experiments, we observed that both formulations yielded improved survival over sham treatment (P < 0.01) as determined by log-rank Mantel-Cox test (Fig. 6). The Ag-PPE-SCKs and SCC1 resulted in survival advantage of 58% and 50%, respectively compared with sham treatment. While the DEM generally would predict a slightly higher efficacy of SCC1 over the Ag-PPE-SCKs, the nanoparticles yielded slightly higher survival *in vivo* despite only being dosed twice 24 hours apart (Fig. 6). Even though we were unable to calculate the median survival hours for the Ag-PPE-SCK-treated mice for direct correlation with the DEM due to greater than 50% survival, the observation that at least 50% of mice in this cohort were alive at 54.2 hours validates the predictive ability of the DEM.

### A dose response is observed for survival with SCC22, but not SCC1, in *P. aeruginosa* infected mice

**Figure 7:**
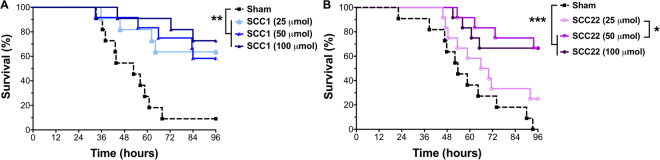
Dose-response studies performed using DMSO-free formulations of (**A**) SCC1 and (**B**) SCC22, where *P ≤ 0.05; **P ≤ 0.01; ***P ≤ 0.001 as determined by log-rank Mantel-Cox test. Six mice were used per treatment arm for each experiment and the data shown are pooled from two independent experiments.

SCC1 and SCC22, due to their highest *in vitro* DEM, that is least toxicity with highest efficacy, and their ability to confer survival advantage in the murine *P. aeruginosa* pneumonia model compared with sham treatment, were chosen for dose-response studies. Both compounds were tested *in vivo* at increasing dosages (25, 50, and 100 µmol), first in the presence of DMSO to further evaluate their antimicrobial efficacy and safety. Surprisingly, with DMSO in the solution, higher doses of SCCs exhibited lower survivals than lower doses of these compounds. For instance, with SCC1, the lowest dose (25 µmol) resulted in a 42% survival advantage; whereas, the highest dose (100 µmol) resulted in the lowest survival advantage of 17% compared with sham treatment (Supplementary Figure S2A). However, each dose provided a significant survival advantage over sham treatment (P ≤ 0.001). Similarly, SCC22 treatment at 25 and 50 µmol led to 58 and 50% survival advantage, respectively (P ≤ 0.05); however, the highest dose (100 µmol) did not yield any survival advantage over sham treatment (Supplementary Figure S2B). Next, these studies were repeated without DMSO in the solution to delineate the effects DMSO may have in this acute infection model when used in combination with SCCs. Interestingly, when the *in vivo* efficacy studies were performed using DMSO-free formulations of SCC1 (Fig. 7A), no definitive dose-response trend could be distinguished and the highest dose led to a slight, but non-significant improvement in survival advantage (100 µmol, 73%) compared to the two lower doses (50 µmol, 64%; 25 µmol, 58%). However, all three doses provided a significant survival advantage in comparison with sham treatment (P ≤ 0.01). On the other hand, when similar studies were performed using DMSO-free formulations of SCC22 (Fig. 7B), a well-defined correlation between therapeutic dose and efficacy was observed with the lowest dose (25 µmol) resulting in a 25% survival advantage and the two highest doses (50 and 100 µmol) providing a 67% survival advantage over sham treatment (P ≤ 0.001).

## Discussion

We, in collaboration with the Youngs and Wooley research groups, have developed a library of silver-based antimicrobial formulations including silver carbene complexes (SCCs) and silver-bearing biodegradable nanoparticles in response to the rapidly shrinking antimicrobial pipeline. In this work, we describe the *in vitro* drug efficacy metric (DEM) and demonstrate its superior correlation with *in vivo* survival for a subset of SCCs compared with other *in vitro* efficacy and toxicity parameters, thereby highlighting its utility in identifying efficacious yet safe silver-based antimicrobials for further studies. Further, we demonstrate that treatment with aerosolized Ag-PPE-SCKs, which have an *in vitro* DEM and therefore predicted *in vivo* efficacy comparable to SCC1, indeed provides a survival advantage comparable to SCC1 treatment. To our knowledge, the DEM described here is not only the first example of an *in vitro* metric that correlates very strongly with *in vivo* outcomes for a class of antimicrobials, but also the first example of a predictive correlation that has been developed for aerosolized antimicrobials. Furthermore, the assays used for the DEM are amenable to high throughput screening and would allow the rapid screening of a large library of compounds.

A few previous reports document *in vitro* metrics used to predict therapeutic indices for screening potential antimicrobial agents, particularly antimicrobial peptides^36–38^. Since these antimicrobials primarily function by damaging cell membranes^39^, the ratio of the minimal haemolytic concentration (MHC) against erythrocytes and the bacterial MIC (MHC/MIC) is a common metric used to predict *in vivo* efficacy^40^. However, this *in vitro* therapeutic index metric has yet to be demonstrated to reliably predict outcomes *in vivo*. In fact, Copple *et al*. state that the *in vitro* TI cannot be used to reliably predict *in vivo* outcomes^41^. Similar difficulties correlating *in vitro* cellular and bacterial toxicities of topical antimicrobials with *in vivo* efficacies have also been reported^42–45^. Recently, Müller and Kramer defined the term “biocompatibility index” (BI) for antiseptic agents, based on *in vitro* cytotoxicity against L929 murine fibroblasts and the antibacterial activity against *Staphylococcus aureus* and *Escherichia coli*^46^. BI greater than 1 is representative of an antiseptic substance with a more suitable efficacy-cytotoxicity profile than an antiseptic substance with BI less than 1. Even though the authors claim that the BI may more realistically mimic antiseptic action *in vivo* compared with previous indices, the absence of supporting *in vivo* correlative evidence is a shortcoming of this study. Other models relying on *in vitro* data to predict *in vivo* antimicrobial efficacy evaluate bacterial susceptibilities following drug exposure at concentrations predicted based on pharmacokinetics of the agent^47–49^. While useful in certain situations^50^, these models require pharmacokinetic data, which may be unavailable during early phases of drug development.

Our primary goal is to develop the silver-based antimicrobials as aerosolizable therapeutics. Considering the importance of developing indication specific tests with on-target cell types for the evaluation of cytotoxicity of drug candidates^33,46^, we initially performed cytotoxicity testing of SCCs using 16HBEs. We considered the use of primary tracheal epithelial cells grown at air-liquid interface; however, we have previously shown that the LD_50_ of primary cells exposed to SCC1 is about 2 log_10_ higher than that of 16HBE such that the amount of drug required for screening would have rendered the study impractical^51^. Secondary cytotoxicity studies with human fibroblasts were performed, not only because fibroblasts are commonly used for cytotoxicity studies, but also because we desired to explore the effect that an off-target cell type on the predictive capabilities of the DEM. The low toxicity of SCC1 with its methylated caffeine backbone is not surprising considering such xanthine derivatives have low toxicity and have been used for multiple purposes including bronchodilation^52^. Of the other three SCCs, which are all based on a 4,5-dichloroimidazole backbone, SCC23 and SCC28 are the most toxic, potentially due to the incorporation of naphthalene functionalities, a structural modification that was performed to increase hydrophobicity. The higher toxicities with these SCCs point towards the effect of their backbone substituents and suggest that by choosing safe carrier molecules, enhanced activity can be obtained while ensuring low toxicity.

The four SCCs investigated in this work demonstrated comparable potency based on their MICs and MBCs, as previously described. These results suggest that the antimicrobial activity is primarily due to the bioactive Ag^+^ cation and the carrier molecules appear to contribute minimally to antimicrobial activity. Next, we calculated the DEMs for each cell type using both 16HBEs and HDFs. As with the LD_50_ measurements, an excellent separation between the SCCs was observed when using 16HBEs, but a minimal separation was noted when using HDFs. Thus, based on the DEM data for 16HBEs, SCC1 and SCC22 appear to possess the best balance of efficacy versus cytotoxicity among the four SCCs; whereas, no such distinction can be made based on the DEM data for HDFs. Unfortunately, because of the comparable potency of all four SCCs against PA M57-15, the LD_50_ values appear to overly drive the DEM calculations, which is a drawback of this study.

The survival advantage observed in our murine *P. aeruginosa* pneumonia model following aerosolized SCC1 and SCC22 treatments, but not SCC23 and SCC28 treatments (Fig. 3), despite the excellent *in vitro* potency of SCC23 and SCC28, suggests a strong effect of drug toxicity during infection. To further elucidate this observation and determine whether the toxicity was localized to the lung tissue, we examined the lung tissue of PA M57-15 infected mice following SCC treatments. Histological examination of the lung tissue following concomitant SCC exposure in *P. aeruginosa* infected mice demonstrated a complete absence of bacterial colonies in all but sham-treated mice suggesting that all four SCCs exerted antimicrobial activity *in vivo* (Fig. 4). The superiority of SCC22 observed during survival studies was also reflected during histology studies. The lungs of SCC22 treated mice exhibited the lowest aggregate histopathological score suggesting a lower overall host-response possibly due to a combination of antimicrobial efficacy and low toxicity. Surprisingly, despite the low toxicity of SCC1 *in vitro* and the successful outcome observed with SCC1 treatment in our infection model, the lung tissue of these mice had a histopathological score comparable to the lung tissue of SCC23 and SCC28 treated mice. Furthermore, a lower degree of severity of host-response parameters was observed for SCC23 and SCC28 treatment compared with sham treatment, even though the survival outcome for these two treatments was worse. Overall, these results suggest a poor correlation between *in vivo* efficacy and histological data. A plausible explanation for the observations with SCC23 and SCC28 treatment could be due to the occurrence of additional downstream toxic effects induced by these compounds. While biodistribution studies performed using radiolabelled Ag^+^ show minimal accumulation of SCC1 and Ag-PPE-SCKs in the liver^34^, there is a likelihood that use of a different backbone, such as naphthalene-bearing, may alter the biodistribution of the SCCs and lead to its accumulation in the hepatic tissue causing toxicity, in addition to that directly observed in the lungs.

A Cox proportional hazards model demonstrates 16HBE DEM (R^2^ = 0.5166) to be the best predictor of survival followed by MIC (R^2^ = 0.5014). However, a major limitation of this model is that the hazard ratio (probability of death) is not dependent on time^53^. For instance, an increase in one order of magnitude 16HBE DEM and MIC reduces the log hazard rate by 0.0335 and 2.0282, respectively. Thus, this model merely demonstrates 16HBE DEM to be a better predictor of survival compared with other *in vitro* parameters, but fails to provide any insights into the effect of *in vitro* parameters on the survival time. This limitation was addressed by correlating the DEM to survival times using linear regression.

The correlation between *in vitro* efficacy and toxicity parameters including the combined DEM metric with *in vivo* efficacy exhibited a varied response (Fig. 5). While MIC, and not MBC, alone demonstrates a robust correlation with *in vivo* efficacy, both these parameters demonstrate a correlation in reverse order with survival. Therefore, even though lower MIC and MBC values indicate superior *in vitro* potency, they necessarily do not translate to *in vivo* efficacy. For instance, the MIC correlates the best statistically with survival hours (R^2^ = 0.9753); however, this correlation fails to actually show an *in vivo* response as expected. Based on the MIC vs. efficacy correlation, SCC23 and SCC28 should perform the best and result in the highest survival of mice; however, SCC23- and SCC28-treated mice exhibit a survival outcome that is worse than sham-treated animals. The LD_50_ for on-target 16HBEs demonstrated a better correlation with *in vivo* efficacy compared with the LD_50_ for off-target HDFs, yet the LD_50_ values for both cells demonstrated a direct correlation with survival, suggesting lower *in vitro* toxicity is indicative of a superior *in vivo* outcome. The combined DEM metric based on 16HBEs (R^2^ = 0.9932) demonstrates the second-best correlation with median survival hours along with a minor statistical difference compared with MIC. However, with regards to actual biological antimicrobial efficacy, this metric, which considers both the safety and efficacy of the drug candidates against an on-target cell type, exhibits the best correlation since the antimicrobial candidates with the highest DEM also exhibit the highest median survival hours. Similarly, preliminary studies also demonstrate a robust correlation between *in vivo* efficacy and the DEM when replacing PA M57-15 with another strain of *P. aeruginosa* (PA O1) for antimicrobial studies (data not shown). In contrast, the correlation with survival hours considerably worsened when using the same metric based on HDFs (R^2^ = 0.0595). These findings are noteworthy, not only because they validate our hypothesis, but also because of the distinction they provide between our DEM metric and the BI proposed by Müller and Kramer^46^. Even though both metrics are defined using essentially the same parameters (DEM = LD_50_/MBC vs. BI = IC_50_/MBC), the BI metric would have falsely predicted SCC23 and SCC28 to be safe since each SCC would have a BI > 1 (based on both 16HBE and HDF cytotoxicity data). In fact, based on BIs determined using off-target cells such as HDFs, SCC23 would have been incorrectly identified as a lead candidate, which in turn would have led to an extremely poor prediction of *in vivo* efficacy. Thus, an appropriate metric coupled with a judiciously chosen on-target cell type is critical for identifying efficacious, yet safe compounds.

One of our main goals was to identify *in vitro* parameters capable of predicting *in vivo* efficacy. Considering the excellent correlation between the *in vitro* 16HBE-based DEM and *in vivo* efficacy for the SCCs, we sought to explore whether the DEM would predict efficacy of another silver-based antimicrobial formulation, namely Ag-PPE-SCK NPs. Since both SCC1 and Ag-PPE-SCKs demonstrated comparable *in vitro* DEM values, they were compared head-to-head in our acute *P. aeruginosa* infection model and we observed a slightly better than predicted outcome with the Ag-PPE-SCKs (Fig. 6). The most likely reason for this outcome is the different pharmacokinetic profiles of SCCs, which distribute through the body quickly, and the nanoparticles, which reside in the lungs for longer periods of time and therefore provide a sustained delivery of the encapsulated SCC at the target site^35^. Because the DEM relies on a balance of cytotoxicity and efficacy in predicting *in vivo* efficacy without taking pharmacokinetics into account, the predictive capability is not as robust as with the SCCs. Thus, the data also point towards the importance of detailed pharmacokinetic (PK) studies as the essential next step in the development of new drug candidates and their formulations.

Our initial *in vivo* studies utilized DMSO for all SCC formulations owing to the lipophilic nature of SCC23 and SCC28 and the need to maintain consistency across all formulations, yet SCC1 and SCC22 are hydrophilic and do not require DMSO for solubilisation. Further, the presence of DMSO in our current studies led to a lower survival for infected SCC1-treated mice at similar doses (25 μmol) in the same acute lung infection model, compared to our previously published observation^21^. Additionally, the distinct survival advantage conferred using SCC1 and SCC22 even at a low dose (25 μmol) in the initial *in vivo* experiments sparked the motivation for the dose-response studies. Our goal for this set of experiments was to explore (a) the effects of DMSO on the efficacy of SCCs in our acute infection model and (b) the dose-response of SCC1 and SCC22. When comparing the efficacy of DMSO-containing (Figure S2) and DMSO-free (Fig. 7) SCC formulations at three doses (25, 50, and 100 μmol), each delivered by nebulization to *P. aeruginosa* infected mice, the use of DMSO appears to have more of an adverse effect on survival with SCC1 than SCC22. Further, in the presence of DMSO, higher doses of SCCs provide less therapeutic benefit than lower doses, and the differences in survival at higher doses are more pronounced for SCC22 than SCC1. These data indicate that DMSO artificially lowers the efficacy of both SCC1 and SCC22. DMSO, which is a known promoter of drug absorption^54,55^, may have led to an accelerated transport of SCCs from the lung epithelium into the systemic circulation causing a reduction in its local therapeutic potential. Furthermore, Pesanti and Nugent have demonstrated a dose-dependent inhibition of *P. aeruginosa* clearance in intratracheally infected mice following intraperitoneal administration of DMSO^56^. In addition, their data revealed that even a 10% solution of DMSO delivered intranasally, resulted in a detectable increase in susceptibility of mice to *P. aeruginosa* infection^56^. Even though the precise mechanisms related to this phenomenon are unknown, its occurrence may partially explain the observed reduction in efficacy of SCC1 and SCC22 when using DMSO. Furthermore, we speculate that both the transport- and clearance-related effects of DMSO may have contributed to the failure of SCC23 and SCC28 treatments *in vivo* in addition to their confirmed toxicity. Separately, both SCC1 and SCC22 provide a significant survival advantage over sham treatment and a distinct dose-response is observed for SCC22 but not SCC1, in the absence of DMSO. Interestingly, the only significant difference in survival is between the lowest dose of SCC22 and the two higher doses of SCC22 and the three doses of SCC1. Possibly, at higher doses, sufficient SCC22 is present in the lung to maintain therapeutic concentrations considerably higher than the MBC at all times. However, at the lowest dose, the SCC22 concentration may peak following treatment and gradually return to MBC or sub-MBC levels at later time-points, leading us to believe that the transport rate across the lung epithelium, as well as lung-retention of these compounds, may be different. This topic will form the basis of a separate investigation.

In summary, we have shown a robust correlation between the DEM derived using an on-target cell type and *in vivo* efficacy for aerosolized silver-based antimicrobials against *P. aeruginosa* as a model pathogen. While other parameters of *in vitro* efficacy and toxicity demonstrated a moderate-to-good correlation with *in vivo* efficacy, the correlation with the 16HBE-based DEM was the best, which validates our hypothesis. Surprisingly, histological data from the same *P. aeruginosa* infection model used for the survival studies, failed to correlate with the survival outcomes and provide additional insights. Our data also demonstrated the predictive ability of the DEM, as it was successfully able to project the survival outcome following aerosolized administration of a novel silver-bearing Ag-PPE-SCK NP antimicrobial formulation to *P. aeruginosa* infected mice. Lastly, a substantial survival advantage compared with sham treatment was observed in the dose response studies performed using SCC1 and SCC22, compounds with the best collective safety-efficacy profiles. Thus, further investigation of high dose SCC1 and SCC22 as therapeutic options may result in efficacious, yet safe interventions for the treatment of *P. aeruginosa* infections including pneumonia.

## Methods

### Silver Carbene Complexes (SCCs) and Silver-loaded Polyphosphoester Shell-Crosslinked Knedel-like Nanoparticles (Ag-PPE-SCKs)

The SCCs have been synthesized and characterized as previously described^18–20,23,26^. For preparation of aerosolizable solutions, lipophilic SCCs (SCC23 and SCC28) were first dissolved in DMSO (Sigma Aldrich) and then diluted further in distilled-deionized water (DH_2_O), whereas the hydrophilic SCCs (SCC1 and SCC22) were first diluted in DH_2_O, into which DMSO was added. In each sample, the final DMSO concentration was limited to 10% (v/v). PPE-SCK nanoparticles (NPs) were synthesized, loaded with silver, and characterized as previously described^31,35^. Before testing, the nanoparticles were suspended in sterile DH_2_O and sonicated for three 15-second pulses at 50% amplitude to achieve complete resuspension (Misonix Microson Ultrasonic Cell Disruptor XL).

### Bacterial strains, mammalian cells, and growth conditions

*Pseudomonas aeruginosa* isolate PA M57-15 was generously donated by Thomas Ferkol, M.D. (Washington University, St. Louis, MO). PA M57-15 was struck from frozen glycerol stocks on to tryptic soy agar plates (Remel) and incubated overnight at 37 °C in a warm room until individual colonies formed. A single colony was inoculated in 10 mL Mueller-Hinton (MH) broth, grown in a shaking incubator (37 °C, 200 rpm) to log phase (OD_650_ = 0.4), and utilized for experiments after appropriate dilutions. PA M57-15 bacterial suspension at OD_650_ = 0.4 corresponds to 2–3 × 10^8^ CFU/mL, as verified by repeated serial dilution and plating.

16HBEs (16HBE14o-), generously provided by Dr. D. Gruenert (University of California, San Francisco, CA) are a human bronchial epithelial cell line transformed with SV40 large T-antigen using the replication-defective pSVori plasmid. 16HBEs were used as a model to determine the relative toxicities of the SCCs to airway cells^57^. Cells between passages 20 to 35 were cultured in Minimum Essential Medium with Earle’s Balanced Salts (Sigma Aldrich) and non-essential amino acids supplemented with penicillin-streptomycin (1%), L-glutamine (1%), and foetal bovine serum (10%). Additionally, primary human dermal fibroblasts (HDFs) were also utilized for *in vitro* toxicity studies. HDFs at passage 4 were generously donated by Carol Tamminga, M.D. (University of Texas Southwestern Medical Center, Dallas, TX). HDFs were cultured in Dulbecco’s Modified Eagle Medium (Sigma Aldrich) supplemented with penicillin-streptomycin (1%), L-glutamine (1%), and foetal bovine serum (10%) and were utilized between passage 7 to 14 for *in vitro* toxicity studies. 16HBE and HDF cultures were assessed for bacterial contamination visually, while mycoplasma contamination was assessed both visually and through a PCR-based Universal Mycoplasma Detection Kit assay (ATCC^®^ 30-1012K^TM^).

### Determination of minimum inhibitory and bactericidal concentrations (MICs and MBCs)

The MIC and MBC determinations of the various SCCs and the Ag-PPE-SCK NPs were performed using a standard CLSI protocol with minor modifications. A mucoid clinical isolate of *Pseudomonas aeruginosa*, PA M57-15, from a cystic fibrosis patient was utilized, because we have previously studied this strain in murine models of *P. aeruginosa* lung infection^21,29,30^. PA M57-15 grown to log phase as previously described was diluted to a density of 10^6^ CFU/mL in MH and added 1:1 in a 96-well plate with different concentrations of SCCs dissolved in 95:5 (v:v) DH_2_O:DMSO solution or the Ag-PPE-SCKs suspended in DH_2_O. The final SCC concentrations tested were 0.25, 0.5, 0.75, 1, 1.5, and 2 μg/mL and were increased in two-fold increments from 2 μg/mL to 32 μg/mL. The final DMSO concentrations in the wells was 2.5% (v/v) and had no impact on bacterial growth, as determined by growth in the positive control wells. The plates were incubated at 37 °C for 18–24 hours, after which the MIC was determined as the lowest silver concentration that resulted in a lack of growth (clear wells) as determined by visual inspection. Contents of these clear wells were streaked on agar plates and incubated for an additional 24 hours at 37 °C. The MBC was determined as the lowest silver concentration that resulted in no bacterial growth on agar plates. Three technical replicates were employed for each experiment and a total of three biological replicates were performed on separate days (3 technical replicates x 3 biological replicates = 9 total replicates) to account for day-to-day variations in media, bacterial growth, compound dilutions, as well as other experimental conditions. The MIC and MBC values that were identical for at least two readings out of the three are reported.

### *In vitro* LD_50_ determination by alamarBlue^®^ and calculation of the Drug Efficacy Metric (DEM)

16HBEs and HDFs grown to confluency (90–95%) as described above were sub-cultured, re-suspended in the appropriate medium, and seeded in wells of clear 96-well plate(s) at either 25,000 cells/well (16HBEs) or 15,000 cells/well (HDFs) with 100 µL of feeding media and incubated over 24 hours at 37 °C, 5% CO_2_, and 100% RH. The cells were then washed once with sterile phosphate buffered saline (PBS, pH = 7.4) and 100 µL of SCCs or the Ag-PPE-SCKs at various concentrations in OPTI-MEM^®^ (Invitrogen) with 3.3% (v/v) DMSO, or without for the Ag-PPE-SCKs, was added to the cells. Next, 10 µL of alamarBlue^®^ (Invitrogen) was added to each well and the plates were re-incubated for 24 h. Finally, the plates were measured for optical density at 570 and 600 nm with a spectrophotometer (POLARstar Omega, BMG Labtech or Cytation 5, BioTek). Untreated cells and media only (with alamarBlue^®^) were used as controls. Initial testing was performed to ensure that both cell lines were viable in OPTI-MEM^®^ over 24 hours without any adverse effects and the presence of DMSO at the concentrations used in our studies did not affect the cytotoxicity results. The relative viabilities of the cells were determined as per the manufacturer’s instructions and using GraphPad Prism^®^ software to generate a non-linear curve fit and interpolate the *in vitro* LD_50_ for each SCC. This assay was performed with three technical replicates for each SCC and Ag-PPE-SCK dose and biological replicates were performed in quadruplicate on separate days (3 technical replicates × 4 biological replicates = 12 total replicates). The DEM for each SCC was determined as the ratio of the molar LD_50_ and MBC concentrations (LD_50_/MBC).

### Murine *P. aeruginosa* acute lung infection model

Weight-matched male C57BL/6 J mice aged 6–8 weeks were used for all acute lung infection studies, which were approved by the University of Texas Southwestern Medical Center Institutional Animal Care and Use Committee (IACUC). All experiments were performed in accordance with relevant guidelines and regulations. Animals were housed in a barrier facility under pathogen-free conditions until they were inoculated with bacteria. The previously described clinical mucoid strain of *Pseudomonas aeruginosa* (PA M57-15) grown to mid-log phase (OD_650_ = 0.4) in Luria broth (LB) was used for all pulmonary infection experiments. The density of the bacterial culture (CFU/mL) was determined using serial dilution and plating for each experiment.

Mice were anesthetized by intraperitoneal administration of a ketamine (60 mg/kg) and xylazine (8 mg/kg) cocktail, intranasally inoculated using 75 µL of the bacterial inoculum (~1 × 10^6^ CFU per mouse), and assigned randomly into treatment groups. Treatment was administered at 1-hour post-inoculation and again every 12 hours thereafter up to a 5th dose at 49 hours *via* nebulization of 5 mL of SCC solutions (containing 25 µmol total Ag^+^ cation per dose for each SCC) or sham treatment. For the head-to-head comparison of unencapsulated SCC1, SCC22, SCC23, and SCC28, all SCCs were dissolved in a 90:10 (v/v) mixture of DH_2_O and DMSO; whereas, the sham treatment consisted of 90:10 DH_2_O: DMSO, but with dilute phosphate buffer (1 mM Na_2_HPO_4_, 0.2 mM KH_2_PO_4_) incorporated to provide tonicity for nebulization. For the dose-response studies with unencapsulated SCC1 and SCC22, 5 mL of SCCs dissolved in DH_2_O alone or 90:10 (v/v) mixture of DH_2_O and DMSO was nebulized to provide a total dose of 25, 50, or 100 µmol Ag^+^ cation per dose for each SCC. Nebulization of the SCCs was achieved using a previously described experimental setup comprising of an Aeroneb Lab apparatus connected to a multi-dosing chamber^58^. Throughout each study, the mice were monitored constantly and times of death were recorded. Every 24 hours, the mice were weighed and assigned cumulative clinical illness scores (0–6) based on their resting posture (0–2), condition of their fur (0–1), and overall activity level (0–3), with higher scores indicating more severe illness^30^. The investigators assigning the scores were not blinded to the treatment group, which is a limitation of this study. The survival studies were all performed in duplicate with results pooled.

*In vivo* efficacy testing of the silver loaded, polyphosphoester, shell-crosslinked, knedel-like nanoparticles (Ag-PPE-SCKs) was carried out in a similar manner as with the SCCs. Ag-PPE-SCKs were suspended in 5 mL DH_2_O to a concentration of ~5 mM silver, as previously determined by mass spectrometry, and empty PPE-SCKs were suspended corresponding to the same mass of polymer. SCC1, a comparator treatment, was similarly dissolved to 5 mM in 5 mL DH_2_O, and sham consisted of the DH_2_O:phosphate buffer solution used in the other studies. The infection model was carried out as before; however, the Ag-PPE-SCKs and PPE-SCKs were dosed at 1 and 25 hours post-infection with the nanoparticles and given sham treatment at 13, 37, and 49 hours. SCC1 and sham treatment groups were dosed at all five time points. Survival studies were performed twice and pooled, though SCC1 was only used in one of the survival studies.

### Histology

Male C57BL/6 J mice were infected with PA M57-15 and treated with the four SCCs or vehicle as described above. At 25 hours, following three treatments, the mice were humanely euthanized by cervical dislocation following anesthetization by a ketamine/xylazine cocktail. Lungs of the mice were harvested aseptically, the lung sections fixed in 10% formalin for a minimum of 12 hours, and embedded in paraffin using an automated rapid tissue processor (Peloris-Leica Microsystems Inc., Buffalo Grove, IL). Two 5-micron thick sections were obtained from each paraffin block and stained with Haematoxylin/Eosin on an automated stainer (Symphony, Ventana-Roche, Tucson, AZ). Sections were evaluated by light microscopy (Nikon Eclipse-Ni, Nikon Instruments, Inc., Americas), scored using a semi-quantitative scale from 0 (negative), +1 (mild), +2 (moderate), and +3 (severe) for the following parameters: inflammation, consolidation, congestion/haemorrhage, and an aggregate score was calculated for each group^59^. The presence of bacterial colonies if any was also noted. A pathologist, who was blinded to the treatment received by the mice, performed the examination and analysis of tissues. A total of two mice were used in each treatment group.

### Statistical analysis

All analyses were performed using Prism 6 (GraphPad Software, Inc., San Diego CA), with the exception of Cox proportional hazards regression model, which was performed using R version 3.1.2 (2014 The R Foundation for Statistical Computing, Vienna Austria). A Cox proportional hazards regression model fitted on the survival time (censoring time at 96 h) with treatment as the independent variable was performed to identify the *in vitro* parameter (MIC, MBC, 16HBE LD_50_, HDF LD_50_, 16HBE DEM, or HDF DEM) that demonstrates the highest correlation with survival *in vivo*. In addition, statistical differences among DEMs were determined using ordinary one-way ANOVA with Tukey’s multiple comparisons test and data were determined to be significantly different for p ≤ 0.05. The *in vivo* survival curves in the infection model were compared using a Log-rank Mantel-Cox test; whereas, differences in weight change and clinical scores of mice receiving different treatments were analysed using Kruskal-Wallis one-way ANOVA and Dunn’s multiple comparison test.

## Electronic supplementary material

Supplementary Dataset 1
