# Supplementary material for: A novel in vitro metric predicts in vivo efficacy of inhaled silver-based antimicrobials in a murine Pseudomonas aeruginosa pneumonia model
Source: Sci Rep. 2018 Apr 23;8:6376. doi: 10.1038/s41598-018-24200-w (PMC5913254; doi:10.1038/s41598-018-24200-w)
Supplement: Supplementary file 1 — Supplementary Dataset 1 [file 41598_2018_24200_MOESM1_ESM.pdf]

A novel *in vitro* metric predicts *in vivo* efficacy of inhaled silver-based antimicrobials in a murine *Pseudomonas aeruginosa* pneumonia model

Parth N. Shah,[#1] Kush N. Shah,[#1] Justin A. Smolen,[2] Jasur A. Tagaev,[3] Jose Torrealba,[4] Lan Zhou,[6] Shiyi Zhang,[2] Fuwu Zhang,[2] Patrick O. Wagers,[5] Matthew J. Panzner,[5] Wiley J. Youngs,[5] Karen L. Wooley,[2] Carolyn L. Cannon[1,*]

[1] Department of Microbial Pathogenesis and Immunology, Texas A & M Health Science Center, College Station, TX 77843, United States.

[2] Department of Chemistry, Department of Chemical Engineering, Department of Materials Science and Engineering, and Laboratory for Synthetic-Biologic Interactions, Texas A & M University, College Station, TX 77842, United States.

[3] Tashkent Paediatric Medical Institute, Tashkent, Uzbekistan, 100140.

[4] Department of Pathology, University of Texas Southwestern Medical Center, Dallas, TX 75390, United States.

[5] Department of Chemistry and Center for Silver Therapeutics Research, The University of Akron, Akron, OH 44325, United States.

[6] Department of Statistics, Texas A & M University, College Station, TX 77842, United States.

[*] Corresponding Author:
Carolyn L. Cannon, MD, PhD
Email: cannon@medicine.tamhsc.edu
Phone: (979) 436-0868; Fax: (979) 845-3479

[#] Authors with equal contribution

<u>**Supplementary Data**</u>

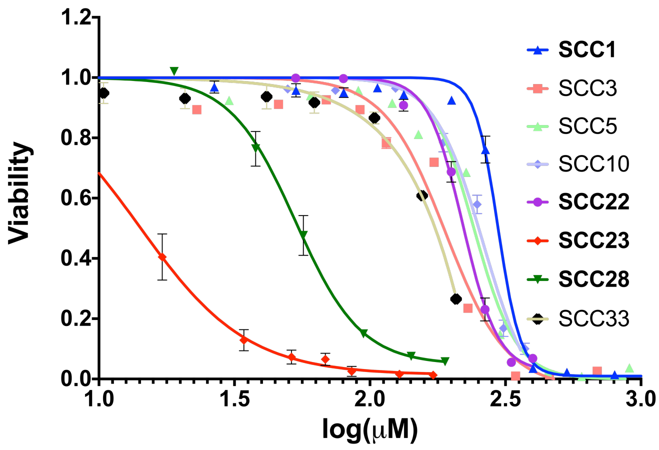

Figure S1. Cell viability curves of eight SCCs (out of a library of ~30 SCCs) against 16HBE cells determined using an alamarBlue® assay.

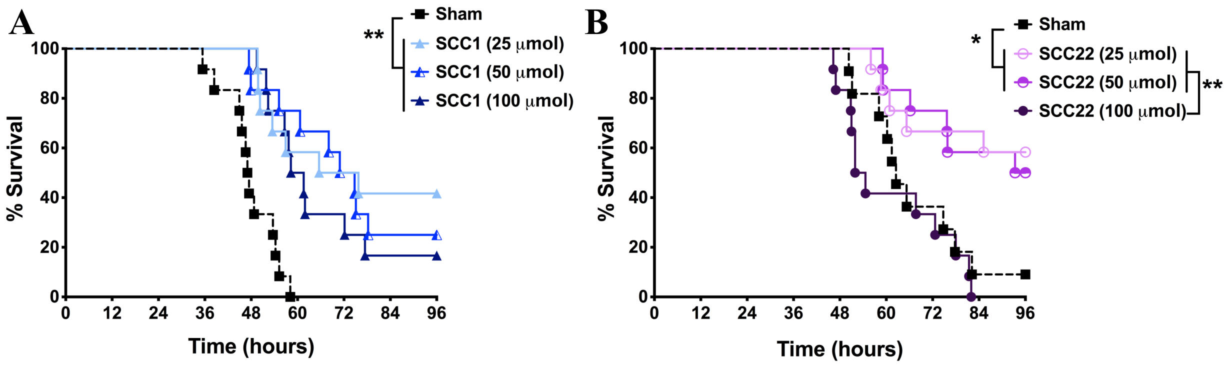

Figure S2. Dose-response studies demonstrating mouse survival post-infection with *P. aeruginosa* strain PA M57-15 and after treatment with various doses of (**A**) SCC1 with DMSO and (**B**) SCC22 with DMSO. * P ≤ 0.05; ** P ≤ 0.01 as determined by log-rank Mantel-Cox test.
